# Maternal Factors that Induce Epigenetic Changes Contribute to Neurological Disorders in Offspring

**DOI:** 10.3390/genes8060150

**Published:** 2017-05-24

**Authors:** Avijit Banik, Deepika Kandilya, Seshadri Ramya, Walter Stünkel, Yap Seng Chong, S. Thameem Dheen

**Affiliations:** 1 Department of Anatomy, Yong Loo Lin School of Medicine, National University of Singapore, Singapore 117594, Singapore; antab@nus.edu.sg (A.B.); e0001953@u.nus.edu (D.K.); a0123640@u.nus.edu (S.R.); 2 Singapore Institute of Clinical Sciences, A*STAR, Singapore 117609, Singapore; walter_stunkel@sics.a-star.edu.sg; 3 Department of Obstetrics and Gynaecology, Yong Loo Lin School of Medicine, National University of Singapore, Singapore 119228, Singapore; yap_seng_chong@nuhs.edu.sg

**Keywords:** epigenetics, neurodevelopmental disorders, attention-deficit hyperactivity disorder (ADHD), maternal factors, fetal development, lifestyle

## Abstract

It is well established that the regulation of epigenetic factors, including chromatic reorganization, histone modifications, DNA methylation, and miRNA regulation, is critical for the normal development and functioning of the human brain. There are a number of maternal factors influencing epigenetic pathways such as lifestyle, including diet, alcohol consumption, and smoking, as well as age and infections (viral or bacterial). Genetic and metabolic alterations such as obesity, gestational diabetes mellitus (GDM), and thyroidism alter epigenetic mechanisms, thereby contributing to neurodevelopmental disorders (NDs) such as embryonic neural tube defects (NTDs), autism, Down’s syndrome, Rett syndrome, and later onset of neuropsychological deficits. This review comprehensively describes the recent findings in the epigenetic landscape contributing to altered molecular profiles resulting in NDs. Furthermore, we will discuss potential avenues for future research to identify diagnostic markers and therapeutic epi-drugs to reverse these abnormalities in the brain as epigenetic marks are plastic and reversible in nature.

## 1. Introduction

Neurodevelopmental disorder (ND) is a collective term that denotes disorders resulting in abnormal brain development at the neonatal stage and cognitive impairment. This includes both structural defects such as neural tube defects (NTDs), and neuropsychological deficits such as impairments in motor and sensory organization, delayed speech and language, difficulties in learning, and other social interactions. These impairments and defects can either be fatal and/or disabling, affecting the children’s quality of life [1].

There is a wide range of NDs detected in newborns and adults, including Attention-Deficit Hyperactivity Disorder (ADHD), autistic spectrum disorders, epilepsy, Down syndrome, Prader-Willi syndrome, schizophrenia, congenital immunodeficiency-centromeric instability-facial anomalies (ICF) syndrome, Rett syndrome, bipolar disorder, and Tourette’s syndrome [2,3]. Among these, ADHD has been shown to be the most prevalent worldwide. The global prevalence of ADHD was estimated to be 5.29% a decade ago [4]. Data from the National Survey of Children’s Health (NSCH) suggests that an estimated 9.5% of children aged 4–17 years were diagnosed with ADHD in 2007, which was further increased to 11% in 2011 in the United States [5]. A recent meta-analysis of many worldwide studies reveals that one in every 20 children is diagnosed with ADHD [6]. It is pertinent to mention that the psychological and physical impairments of most NDs take a massive toll on individuals and pose psychological and economic burdens on the family and society.

The genetic etiology for most NDs is still not clear. Although some disorders have been linked to specific mutations in single or multiple genes, there are many caused by varied factors such as genetics, epigenetics, and in*-*utero environment, in mothers as well as fetuses. While genomic variations such as chromosomal deletions/duplications and/or single-nucleotide mutations/polymorphisms could be either inherited or de novo in offspring, non-heritable genetic alterations are largely linked to epistatic and epigenetic alterations during development [7]. It is well established that the in*-*utero environment plays a vital role in embryogenesis. There are several factors such as the physiological and biochemical milieu of the maternal uterus during oocyte maturation, peri-implantation, and post-implantation stages that contribute to epigenetic alterations in the embryonic transcriptome, resulting in abnormal fetal development [8]. The placenta has also been shown to be affected by several maternal factors, leading to abnormal epigenetic regulation of various developmental genes such as LINE-1 and AluYb8, which in turn may result in neurodevelopmental disorders in the offspring [9]. During pre-term deliveries, significantly lower levels of the two most prominent neurotrophic factors, brain-derived neurotrophic factor (BDNF) and nerve growth factor (NGF), were detected in the placental tissue and umbilical cord plasma, suggesting plausible altered epigenetic mechanisms that could lead to altered fetal programming [10].

In view of the fact that epigenetic factors contribute to fetal development [11], it is crucial to elucidate the underlying epigenetic mechanisms playing a part in the pathophysiology of these NDs [12,13,14]. Furthermore, the epigenetic mechanisms have been shown to be altered by several factors such as lifestyle, infection, genetic and metabolic alterations, as well as other maternal factors, contributing to the neurological disorders in the offspring [15,16,17,18]. This review will bring forth a comprehensive analysis of the recent findings regarding the epigenetic landscape contributing to the pathophysiology of NDs. As epigenetic alterations are plastic and reversible in nature, potential avenues towards diagnostic and therapeutic strategies for the better management of these disorders are discussed.

## 2. Maternal Factors Contributing to Neurodevelopmental Disorders in Offspring

Several maternal factors have been reported to be detrimental to the neurodevelopmental process in*-*utero [19]. Genetic disorders are one of the most prevalent factors in the etiology of NDs. Down syndrome, Prader-Willi syndrome, Rett syndrome, Fragile-X syndrome, and immunodeficiency-centromeric instability-facial anomalies (ICF) are found to be associated with single gene mutations or chromosomal aberrations, whereas other disorders such as NTDs, ADHD, autism spectrum disorder, epilepsy, Tourette’s syndrome, fetal alcohol syndrome, and schizophrenia are linked to multifactorial etiologies (genetic, epigenetic, and environmental) [1].

Fetal development is known to be affected when pregnant women are exposed to various factors such as malnutrition [20], viral or bacterial infection [21], metabolic disorders [18], obesity [22], smoking and alcohol consumption [23], a high-fat diet [24], and advanced maternal age [25]. However, the exact mechanism by which these environmental cues contribute to NDs is not clear. The type, time, and level of exposure to the above mentioned teratogens and risk factors during fetal development may play a critical role in determining the potential genotypic and phenotypic outcomes in the offspring. 

## 3. Epigenetic Association in Pathophysiology of NDs

Epigenetics mechanisms involving DNA methylation, post-translational modifications of histone proteins, and transcriptional regulation by non-coding RNAs including miRNA, siRNA, piRNA, and lncRNA alter the gene expression pattern without altering the DNA sequence. DNA methylation, catalyzed by the DNA methyltransferases (DNMTs), is a key player in epigenetic silencing of gene transcription, whereas histone modification is a post-translational process whereby histone proteins undergo methylation, acetylation, ubiqutination, sumoylation, and phosphorylation, leading to chromatin modification [26]. There are different classes of histone modifying enzymes such as histone methyl transferases (HMTs), histone demethylases (HDMs), histone acetyltransferases (HATs), and histone deacetylases (HDACs) that control gene expression by modulating their transcriptionally active promoter sites [26]. Furthermore, non-coding RNAs largely regulate gene expression by acting at the post-transcriptional level without coding for any functional proteins [27].

Epigenetic pathways have transformed our understanding of molecular genetics [28]. The DNA CpG methylation blueprints for several developmental genes have been established for the early embryonic stage [29]. During neurulation, neural stem cells (NSCs), the cardinal cells of the central nervous system (CNS), exhibit DNA hyper- and hypo-methylation to support the transcriptional requirements, and neural progenitor cells undergo maturation and cell fate determination by switching off pluripotent genes and switching on lineage-specific genes [30]. Hence the process of early embryonic development in the fetus is susceptible to epigenetic modulation [31,32]. There are numerous reports showing epigenetic linkage in the pathophysiology of NDs. The maternal factors during gestation trigger epigenetic mechanisms that may alter the expression of various genes that play a significant role in fetal development [8]. There are reports demonstrating that epigenetic modulations cause single gene mutations resulting in ND [33,34,35]. In this review, we will summarize the epigenetic imprints that are caused by maternal factors leading to NDs.

## 4. Maternal Lifestyle

Maternal lifestyle factors such as smoking (nicotine and caffeine), alcoholism, and psychosocial stress are reported to be critically associated with epigenetic molecular pathways, leading to abnormal neurological syndrome in childhood. Smoking and alcohol consumption during gestation are two of the most detrimental habits that have been shown to affect language, speech, hearing, and cognitive development in offspring [36]. Children from obese mothers have a higher risk of developing autism spectrum disorders [37]. Meta-analysis data reveal that late maternal age is an associated risk factor for autism in children. Children from mothers aged ≥35 years were found to be more susceptible to autism compared to mothers aged 25–29 years [25]. Animal studies show metabolic-syndrome-like phenotypes in the offspring when mothers were subjected to a high-fat diet during pregnancy. This syndrome was found to be associated with epigenetic alterations in genes important for adipogenesis in newborns [24].

### 4.1. Smoking

Smoking is a leading risk factor for several life-threatening conditions such as cancers and cardiovascular disorders. It has been shown that smoking is associated with aberrant CpG methylation of more than 7000 genes, and for some genes these DNA methylation sites are persistent in the genome even 30 years after quitting smoking [38]. The adverse effects of smoking are not only linked to individuals’ health but also to their child’s development. Maternal exposure to nicotine during pregnancy has been linked to miscarriage, stillbirth, preterm delivery, and several metabolic, cardiovascular, and neurobehavioral disorders in offspring [39]. Maternal smoking is a robust associated risk factor in the etiology of ADHD in children [40]. Continuous Performance Test (CPT) results revealed that children from mothers exposed to nicotine during gestation showed significant attention deficits [41]. Prenatal exposure to nicotine is also associated with the development of Tourette’s syndrome in offspring, with co-morbidities such as tic disorder and obsessive–compulsive disorder [42].

It has been reported that nicotine can cross the placental barrier, leading to epigenetic alterations apparent during the process of fetal developmental [43]. In*-*utero exposure to nicotine disrupts the normal migration of trophoblast cells, leading to placental abruption and difficulties in placental implantation [44]. Recently, several epigenome-wide association studies (EWAS) reported a link between smoking during pregnancy and placental methylation patterns [9,45,46]. These studies have identified various genes depending on their altered methylation and expression pattern.

**Figure 1:**
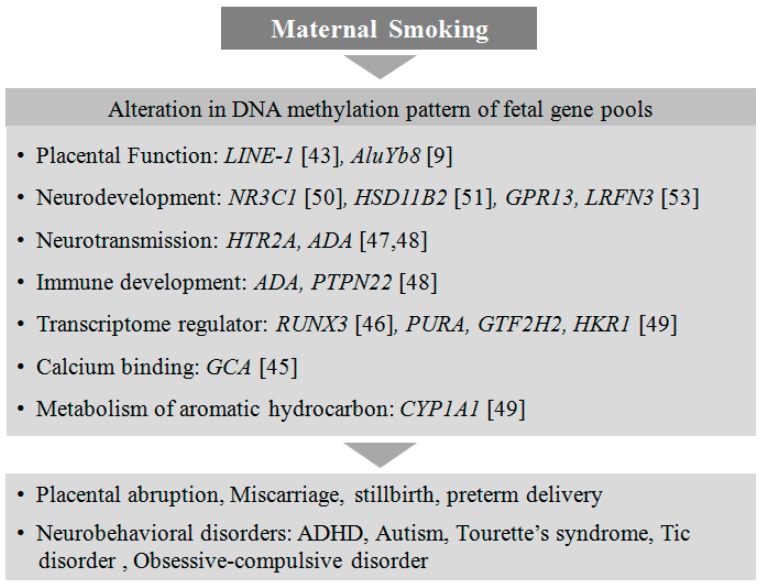
Smoking in mothers alters neurodevelopmental processes in the fetus. Maternal smoking alters the DNA methylation of genes involved in placental and fetal development, leading to neurodevelopmental disorders in the offspring.

Recently, an altered methylation status in the placental serotonin receptor gene *HTR2A* has been shown to be associated with neurobehavioral outcomes and autism in infants, although the intrinsic role of *HTR2A* in neurodevelopment has not been clearly elucidated [47,48]. *CYP1A1* is a catalytic enzyme responsible for the metabolic processing of carcinogenic compounds such as aromatic hydrocarbons present in cigarette smoke. The placental *CYP1A1* gene from mothers exposed to tobacco during pregnancy has been shown to be hypomethylated at CpGs near its promoter [49]. Furthermore, in response to prenatal cigarette exposure, two other genes, *NR3C1*, a glucocorticoid receptor [50], and *HSD11B2*, a corticosteroid dehydrogenase isozyme [51], are found to be epigenetically regulated with hypermethylation in the placenta, associated with poor neurodevelopment in the offspring [52]. There are several differential methylation patterns identified in genes such as *RUNX3* [46], *PURA*, *GTF2H2*, *HKR1* [49], *GCA*, and *GPR13* [53] that are associated with in*-*utero exposure to nicotine. The functions of these genes are not yet well understood in the developing placenta, but they may play important roles in one or the other metabolic pathways essential for healthy fetal development. It is crucial to understand how placental epigenetic regulation due to smoking in mothers can alter the neurodevelopmental process in the fetus and subsequently lead to neurobehavioral abnormalities in children (Figure 1).

### 4.2. Alcohol Consumption

Alcoholism is a widespread social and medical issue in the world today. It not only causes several psychiatric and health-related problems, but is also associated with multiple birth defects in the offspring of alcoholic parents. Fetal alcohol spectrum disorder (FASD) is a series of neurodevelopmental disorders affecting attention, memory [54], craniofacial development [55], motor function [56], and auditory [57] and language problems [58] in children linked to maternal gestational alcohol exposure. FASD includes different diagnosis such as fetal alcohol syndrome (FAS) with or without confirmed prenatal alcohol exposure, partial fetal alcohol syndrome (pFAS), alcohol-related birth defects (ARDB), and alcohol-related neurodevelopmental disorder (ARND). The prevalence rate of FASD varies from six to 12 per 1000 newborns in Western countries, which is much higher than previous estimates [59,60,61]. 

Although the underlying molecular mechanisms in the pathogenesis of FASD are still under investigation, there are several reports highlighting epigenetic imbalance in fetuses due to maternal alcohol exposure [62,63]. A recent DNA methylation study on a FASD cohort from a Canadian population revealed that 658 methylation sites are significantly altered compared to control subjects. Furthermore, several genes found to be hypermethylated were associated with neurodevelopmental disorders [64]. It has also been reported that the timing (stages of gestation) and pattern (binge or continuous) of alcohol exposure play a critical role in the alcohol-induced alteration in the neurodevelopmental process [65,66]. In a mouse study, exposure to acute doses (binges) of alcohol at different trimesters of human-equivalent pregnancy showed differential effects on developmental processes in offspring. Cell proliferation was largely compromised in the first trimester in these mice, compared to cell migration and differentiation in the second trimester and cellular networking in the third [65]. It has been further shown in a rodent model of FASD that prenatal alcoholic exposure during the third trimester resulted in a significant increase in the level of DNMTs, responsible for the repression of gene transcription in the adolescent*.* This in turn resulted in changes in methyl CpG binding protein 2 (MeCP2) expression, essential for the regulation of several genes towards neuronal development and function [67]. Intraperitoneal administration of ethanol in pregnant mice in gestational days 6–8 leads to detrimental morphological malformations in the face, eyes, nose, mandible, vibrissae, and lip palate in 10–15-day-old embryos [68]. The pattern of exposure, both binge-like (acute) and throughout the gestational period (chronic), has a noticeable effect on fetal neurodevelopment. While binge-like exposure incurs maximum damage to the developing fetus such as long-term memory deficits [69] and the altered expression of several developmental genes in the neonatal brain [70,71] as shown in several animal studies, chronic paternal exposure caused epigenetic dysregulation in the mouse neonates [72].

**Figure 2:**
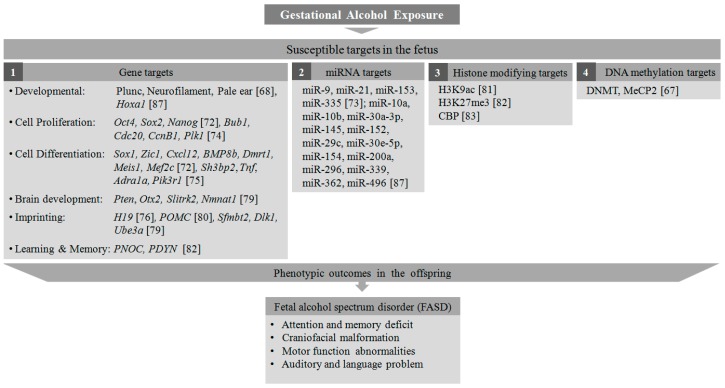
Epigenetic targets of alcohol exposure in the fetus. Gestational alcohol exposure induces histone modification, alteration in DNA methylation pattern and miRNA targets, and expression of genes associated with fetal developmental process, leading to neurodevelopmental disorders.

In a mouse whole-embryo culture study, it has been shown that alcohol exposure induces abnormalities in early embryonic development through altered DNA methylation of several important genes responsible for cell cycle regulation and apoptosis [73]. Mouse embryonic stem cells (mESCs) exposed to ethanol in*-*vitro showed a differential gene expression pattern for several pluripotency-related transcription factors, proliferation, and differentiation markers [72]. During retinoic acid (RA)-induced neural differentiation, the ethanol-treated mESCs exhibited significant upregulation of several transcription factors- *Oct4*, *Sox2*, and *Nanog*, that maintain the pluripotent embryonic stem cell phenotype, suggesting that ethanol exposure maintains cells in their proliferative stage. Furthermore, there was also a striking suppression in differentiation-related genes such as *Sox1*, *Zic1*, *Cxcl12*, *BMP8b*, *Dmrt1*, *Meis1*, and *Mef2c* to derail these cells from RA-directed neuronal fate [72]. Recent reports showed that alcohol exposure leads to hypermethylation of some genes involved in cell cycle regulation such as *Bub1*,*Cdc20*, *CcnB1*,and *Plk1*in neural stem cells [74], and hypomethylation of cell cycle genes involved in neural stem cell differentiation such as *Sh3bp2*, *Tnf*, *Adra1a*, and *Pik3r1* [75]. It has been shown that alcohol can alter the DNA methylation level of important genes such as *H19*, an imprinting gene in the sperm [76] and placenta [77], and *Nr2b*, a glutamate receptor playing a significant role in the formation of long-term potentiation (LTP) [78]. The effect of alcohol teratogenesis in the mouse brain resulted in miRNA-mediated differential methylation in many genes such as *Pten*, *Otx2*, *Slitrk2*, and *Nmnat1*, involved in brain development and functioning. Several imprinting genes such as, *Sfmbt2*, *Dlk1*, and *Ube3a* were also found to be differentially methylated in the mouse brain [79], highlighting the parental links to the pathophysiology of FASD with altered epigenetic pathways in the offspring. Another male germline imprinting gene, *POMC*, has been reported to be hypermethylated with compromised neuronal function in neuronal cells from the offspring of alcohol-exposed rats. The expression levels of histone-modifying proteins and DNA methyltransferases are also found to be altered in these neuronal cells [80]. Furthermore, there are a few reports demonstrating the effect of binge alcohol exposure on the modification of several histone marks. H3K9 acetylation was found to be increased in multiple organs from rats after intragastric delivery of ethanol [81]. The effect of alcohol administration on the amygdala complex in the rat showed a decrease in H3K27 trimethylation and an increase in H3K9 acetylation in the promoter regions of *PNOC* and *PDYN* genes [82]. In a rat model of FASD, acetylation of histones H3 and H4 acetylation was found to be decreased in the developing cerebellar cortex, mediated by the downregulation of CBP, a HAT enzyme [83]. In addition to the histone-mediated transcriptional reprogramming, apoptotic pathways have also been triggered in alcohol-exposed cells. Caspase-3-mediated neural cell apoptosis has been associated with histone modifications such as H3K9 and H3K27 dimethylation in a mouse model of ADHD [84]. The teratogenic effects of alcohol are not only limited to abnormal DNA methylation and histone modification but also alters several miRNAs controlling the expression of many early development control genes [85]. A miRNA microarray study in fetal neural stem cells (NSCs) revealed significant downregulation of miR-9, 21, 153, and 335 upon ethanol exposure [86]. Another microarray study depicts alteration in the expression of a series of miRNAs in the brain tissue derived from a mouse fetus exposed to ethanol in*-*utero. MiRNAs such as miR-9, 10a, 10b, 30a-3p, 145, and 152 were found to be upregulated, while miRNAs such as miR-29c, 30e-5p, 154, 200a, 296, 339, 362, and 496 were downregulated. Among these, the upregulation of miR-10a was associated with downregulation of its putative target gene, *Hoxa1*, essential for embryonic development [87]. From the abovementioned studies it is evident that alcohol-induced epigenetic alteration is a potential hazard to the developing fetus, leading to several neurocognitive and developmental disorders associated with FASD (Figure 2). Although alcohol-induced neurotoxicity can be prevented by stopping maternal exposure to alcohol during pregnancy, a wide range of antioxidants (Vitamin C and E, Resveratrol) have been tried in animals and cell systems to alleviate alcohol-induced neurotoxicity [88]. Other dietary supplements such as folate, choline, and L-glutamine have also been found to be effective in reducing the effects of alcohol in the abnormal neurodevelopmental process [88].

### 4.3. Malnutrition

There is rising concern about the detrimental effects of malnutrition during pregnancy on the developing fetus. The effect of human malnutrition at the prenatal stage is best highlighted during periods of famine during the Dutch Hunger Winter (1944–1945) [89]. The effect of famine significantly contributed to a range of abnormalities in newborns such as congenital neural defects, schizophrenia, and cerebro- and cardiovascular diseases [90,91,92]. Furthermore, several groups have reported that malnutrition during gestation causes epigenetic alteration in the offspring [93,94,95]. Dietary epigenetic regulators are critical in the process of normal fetal development in*-*utero, and an imbalance may cause irreversible phenotypic abnormalities in newborns [96,97]. While diet-induced aberrant DNA methylation in developmental genes is a matter of serious concern, its effects on histone and chromatin modifications and modulation in miRNA expression are largely uncharted. Various dietary methyl group donors such as folate, choline, methionine, betain, and methylcobalamine from our daily diet play critical roles in the epigenetic regulation of DNA methylation through the folate-mediated one-carbon metabolism (FOCM) pathway [98]. Hence, dietary deficiency in any of these micronutrients in the maternal diet may trigger epigenetic alterations in vital developmental genes in*-*utero [17].

The impacts of nutritional and environmental influences on the fetal epigenome were studied in an agouti viable yellow (*Avy/a*) mouse model showing coat color variation, which is correlated with epigenetic markers. The *Avy/a* pregnant mouse model has recently been employed to demonstrate the epigenetic association of methyl-supplemented diet during pregnancy [99]. Furthermore, it has been shown that epigenetic modifications may transgenerationally persist, as the obesity-inducing allele was seen to be inherited from the mother to the offspring in *Avy/a* mice*.* Interestingly, this effect was ameliorated when mothers were fed with a methyl-donor-supplemented diet during pregnancy [100]. Additionally, it has been shown in honeybees that the DNA methylation pattern is differentially altered by different types of honey to determine the queen or worker phenotypes [101,102].

It is well established that maternal folate supplementation during pregnancy is one of the essential factors for normal fetal development [103]. It has been shown that a mutation in the methionine synthase reductase (*Mtrr*) gene, which is functionally responsible for the deployment of methyl groups from the folate cycle, leads to an epigenetic imbalance in the expression of many genes in the placental tissue, as well as abnormal uterine development and congenital malformation like neural tube defects in the newborn [104]. It has been shown in a rodent study that pregnant mothers on micronutrients such as folate and vitamin B12 deficient diet led to downregulation of important genes such as *BDNF*, *CREB*, *NGF*, and *TrkB*, essential for normal brain development and function in the offspring [105]. Furthermore, these genes’ expression could be restored by omega-3 fatty acid supplementation in the maternal diet [105]. Although the direct effect of inadequate dietary supplement on the neurodevelopment of the fetus is not well investigated, there are sufficient reports demonstrating their pivotal role in epigenetic regulation. In our daily diet there are macronutrient derivatives (choline, methionine, betaine) [106,107], micronutrient elements (Vitamin A, D and B) [108,109,110], microminerals (iron, selenium, zinc) [111,112], and bioactive compounds (polyphenols) [113] that have been found to play a significant role in embryonic development through different epigenetic processes.

**Figure 3:**
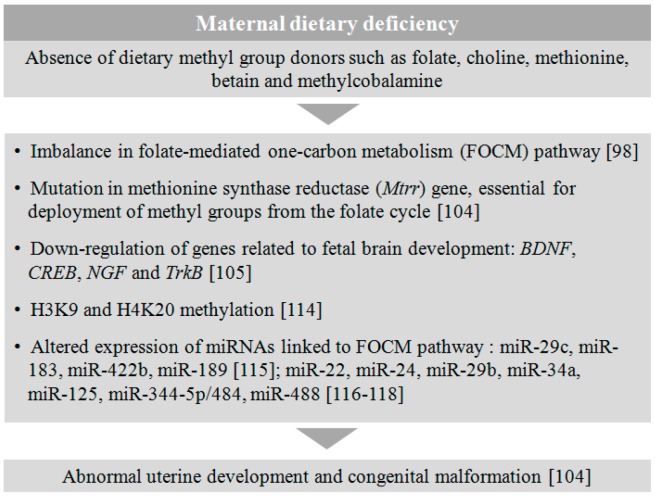
Effect of maternal dietary deficiency on fetal development. The absence of essential dietary supplements in maternal diet during gestation leads to a disruption in metabolic pathways and several epigenetic alterations in the fetus, triggering abnormal uterine development and neurodevelopmental disorders.

Dietary factors have also been shown to regulate epigenetic factors such as histone proteins and miRNAs. The absence or presence of methyl donors in the diet had a direct correlation with the H3K9 and H4K20 methylation in the rat [114]. The effect of maternal nutritional deficiency also significantly altered the expression of several miRNAs targeting genes important for angiogenesis and extracellular matrix remodeling in the offspring [115]. miRNA 29c, 183, and 422b were found to be significantly downregulated and miRNA189 was upregulated in the P1 newborn when mothers were fed on a 50% food-restricted diet [115]. On the other hand, miRNAs such as miR-22, miR-24, miR-29b, miR-34a, miR-125, miR-344-5p/484, and miR-488 were found to be strongly associated with the genes involved in the metabolic regulation of FOCM pathway [116,117,118]. Although there is no substantial direct evidence of dietary influences on the etiology of abnormal embryonic development, the above mentioned reports suggest that there could be a strong association of epigenetic alteration in the offspring from dietary compromised mothers, leading to congenital malformations including neurodevelopmental disorders (Figure 3).

### 4.4. Late Maternal Age and Assisted Reproductive Procedures

It is well established that aging-induced epigenetic changes play a critical role in the etiology of many age-related disorders. The effect of late parental age on fetal development is not well validated in the pre-clinical setup, although there are intermittent reports suggesting epigenetic roles associated with maternal age in the pathophysiology of neurodevelopmental disorders in the offspring [119,120,121,122]. There are several adverse birth events observed in aging mothers, with a larger occurrence of pre-term deliveries. It has been shown that there is increased CpG-methylation at the promoter sites of immunomodulatory genes in human placental tissue during pregnancy, suggesting a role of gestation in pregnancy outcomes [123]. In a recent study, women’s health and late age were found to be significantly associated with hyper-methylation of CpG rich sites in the blood and other tissue DNA from developmental genes [124].

CpG-DNA methylation in the early embryo is susceptible to external factors including maternal health and age at gestation. De novo DNA methylation in germ cells is important in the determination of genomic imprinting and maintenance of pluripotency in the early embryo. During the course of fertilization and implantation, the parental genomes are entirely demethylated before they re-enter the methylation process during germ cell maturation and the methylation status persists until the late stages of fetal development [125].

It is evident that, with increasing age, DNA methylation pattern are compromised in the oocytes, leading to stillbirth and fetal abnormalities [126]. Recently, altered DNA methylation was reported in the aging human sperm targeting promoter regions of 117 genes, among which few are strongly associated with neuropsychiatric disorders [127]. A genome-wide CpG assay in 168 newborn humans showed stronger correlation between differential methylation in the 144 CpG islands associated with 142 genes and maternal age. These genes were found to be associated with several important transcriptional, neurological, and metabolic pathways in the offspring [128].

In addition, maternal age has been found to be one of the risk factors for autism in offspring [129,130]. A meta-analysis of population-based epidemiological studies until 2011 found a 1.5-fold increase in relative risk index for autism in children born to mothers aged above 35 years when compared to mothers between 25–29 years of age [25]. The underlying molecular mechanism in this association is still not clearly depicted, although there are substantial reports linking higher maternal age to negative impacts on the offspring’s health. Age-associated obstetric complications such as the weakening of uterine muscles and reduced blood supply may also add up to associated risks for these neurological disorders in children [131].

With the latest advancement in assisted reproductive technologies (ART), several reproductive strategies such as in*-*vitro fertilization (IVF) with donor egg, sperm, or embryo; intra-cytoplasmic sperm injection (ICSI); superovulation; gamete intra-fallopian transfer (GIFT); zygote intra-fallopian transfer (ZIFT); and surrogacy have become increasingly popular among the population with infertility [132]. Although there is increasing concern about the neurological outcome in offspring from ART, studies available until now could not prove any negative correlation [133,134,135]. A collaborative cohort study on 540 ICSI, 437 IVF, and 538 normal-conception offspring suggests that ICSI and IVF children require more follow-up healthcare compared to normal-conception children, although there were no significant differences in the neurological outcomes between the three groups [133]. Another multi-center cognitive motor assessment study involving 511 ICSI, 424 IVF, and 488 normal conception children concluded that there were no significant differences in the performance IQ (PIQ), verbal IQ (VIQ), and full scale IQ (FSIQ) scores of Wechsler Preschool and Primary Scale of Intelligence-revised (WPPSI-R) and McCarthy Scales of Children’s ability (MCSA) motor scale among these cohorts [134]. There is very little information on ART-induced epigenetic regulation. Recently, super ovulation in mice has been shown to downregulate the expression of miRNAs such as miR-122, miR-144, and miR-211, which are involved in the regulation of neuronal migration and differentiation, linking ART-mediated epigenetic susceptibility to neurodevelopmental disorders [136]. 

## 5. Maternal Metabolic Disorders

**Figure 4:**
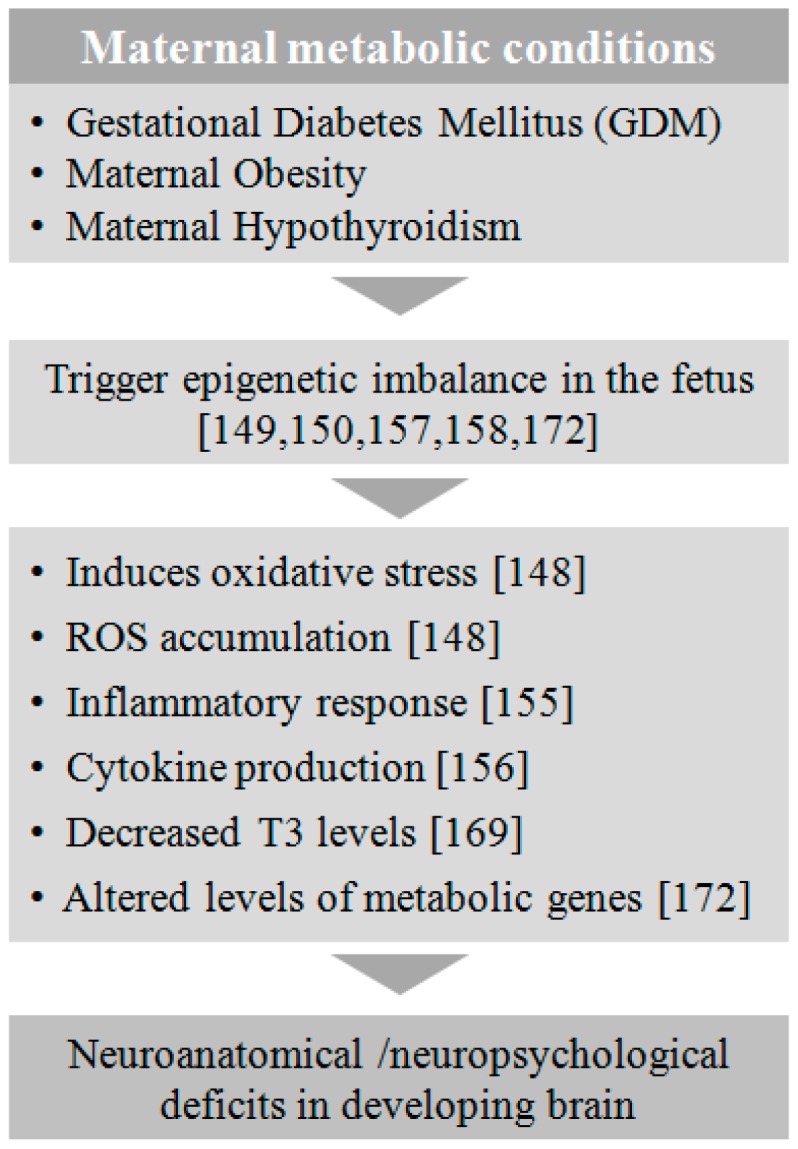
Effect of maternal metabolic conditions on fetal development. Metabolic conditions at gestation such as GDM, obesity, and hypothyroidism induce epigenetic alterations in the fetus, leading to a series of metabolic and immunogenic changes triggering neuroanatomical and neuropsychological deficits in the developing brain.

Maternal metabolic conditions such as diabetes, hypertension, obesity, and thyroidism during pregnancy have been shown to be associated with NDs in children [137] (Figure 4). Several studies have shown that fetuses exposed to altered maternal metabolic conditions are at risk of developing Autism Spectrum Disease (ASD), developmental delays, childhood symptoms of ADHD, eating disorders, and psychotic disorders later in life [137,138,139]. There is collective evidence suggesting that maternal metabolic disorders alter epigenetic mechanisms, which contribute to the neurodevelopmental and metabolic abnormalities in the offspring [140,141,142,143]. Early intervention in the management of metabolic disorders at pre-conception stage is one of the precautionary steps to ameliorate ill effects on the developing fetus.

### 5.1. Gestational Diabetes Mellitus (GDM)

Diabetes, considered a global epidemic, is projected to rise in all age groups, including young adults, from 2.8% in 2000 to 4.4% in 2030 worldwide [144]. This increase in prevalence is due to change in lifestyle, obesity, and inactivity among young adults, which puts women of childbearing age at risk of developing diabetes during pregnancy or before conception [145]. Hyperglycemia, a known teratogen, presents the unborn fetus with an increased risk of developing NTDs or neuropsychological deficits, which imposes an emotional and socioeconomic burden on the family and society. Moreover, maternal diabetes is shown to cause ASD and developmental delay, particularly expressive language deficits in offspring [137]. Recently, many studies have attempted to understand the molecular and epigenetic mechanisms of diabetes-induced brain malformations. The interplay between the environment and epigenome is governed by epigenetic mechanisms that bridge the gap between maternal diabetes and diabetes-induced brain malformations (both anatomical and neuropsychological) in offspring [146]. Since some of these epigenetic changes are heritable [147], this is of serious concern due to transgenerational effects. Thus, early diagnosis and tight control of maternal blood glucose levels are deemed critical for reducing the risk of developing neurodevelopmental disorder in offspring of diabetic mothers. 

It has been shown that maternal diabetes induces oxidative stress and reactive oxygen species (ROS) accumulation [148], which in turn alter the DNA methylation status in the early embryo [149,150]. Furthermore, we have shown the altered expression of several transcription factors and signaling pathways involved in brain development in embryos of diabetic pregnancy in mice, suggesting that maternal diabetes disrupts molecular cues [151,152]. Our group has recently shown that hyperglycemia in pregnant mice perturbs epigenetic mechanisms in NSCs of embryos by altering chromatin organization, histone modifications, DNA methylation, and expression of miRNAs that target genes involved in NSC differentiation [153]. 

In summary, maternal diabetes alters epigenetic mechanisms, which in turn disrupt the expression of many genes involved in neural tube development. It is important to understand how hyperglycemia alters these molecular and epigenetic mechanisms during fetal development as they can have an influence on the neurological function of the offspring in later stages of their life.

### 5.2. Obesity

The frequency of obesity in women has increased from 29.8% in 1980 to 38% in 2013 worldwide [154]. Maternal obesity or excessive weight gain during gestation leads to systemic elevation of fatty acids and glucose along with neurohormones and inflammatory markers [155]. These molecules cross the placenta and enter the fetal circulation, bringing about changes in the neuroendocrine milieu and disturbing the development of neural circuitries [155]. Studies conducted in non-human primates fed with a high-fat diet for 2–4 years before conception resulted in fetallipotoxicity and fatty liver, in turn inducing cytokine production in the fetus. Early exposure to cytokines resulted in perturbation of the serotonergic system of the offspring, eventually leading to behavioral disorders such as anxiety and depression in later stages of life [156].

Alterations in epigenetic mechanisms have been shown to be linked to maternal dietary pattern [157]. An imbalance in maternal nutritional environment affects the enzymes involved in modulations of the fetal epigenome [22]. Mice fed a high-fat diet during pregnancy showed a maternal nutritional imbalance, which led to perturbed embryonic histone acetylation patterns—H3 and H4 and acceleration of growth process in the developing embryo [158].

Histone deacetylase1 (HDAC1), which plays an important role in neurogenesis [159], has been shown to be depleted in the offspring of non-human primate mothers fed with a high-fat diet [160], suggesting a link between neurodevelopment and maternal nutrition-induced epigenetic changes. Furthermore, maternal trends of seasonal food intake were associated with an altered DNA methylation status at metastable epi-alleles (established DNA methylation sites in the genome) in the early embryo. Increased Body Mass Index (BMI) of mothers resulted in decreased systemic DNA methylation in the offspring [161]. Maternal obesity further triggers other metabolic states such as diabetes, thus worsening the outcome. 

Taken together, strict epigenetic coordination is deemed critical during gestation. Any deviation caused by maternal metabolic states results in epigenetic changes that may be among the underlying causes of neuroanatomical or neuropsychological deficits seen in the offspring of obese mothers [162]. Since most of these reports were based on animal model studies, a comprehensive longitudinal study on human development in response to various maternal factors would be advantageous to elucidate the exact mechanism of association between obesity and neurodevelopmental disorders.

### 5.3. Hypothyroidism

Congenital hypothyroidism (CH) is a well-established factor in the etiology of many neurodevelopmental abnormalities in children [163,164,165,166]. Attention deficit is also observed in human subjects suffering from CH [167]. It is further reported that a deficiency of thyroid hormone (TH) in expecting mothers negatively influences fetal brain development [168]. Children from mothers with subclinical hypothyroidism during gestation showed abnormalities in intellectual and motor development at 25–30 months of age [169]. Animal studies also strongly suggest ill effects of CH on brain networking and neurocognitive ability in offspring. Female rats fed on an iodine-deficient diet from the pre-gestation period to the end of lactation showed a significant reduction in T4 hormone in the fetal brain, leading to impaired excitatory synaptic pathways in adulthood [166]. Liu et al. have demonstrated the impairment of long-term memory in seven-day-old rat pups associated with significant downregulation of BDNF in the hippocampus when the mothers were subjected to subclinical hypothyroidism before mating [170]. A recent report shows that abnormal fetal brain development can be ameliorated by administering an HDAC inhibitor in the rat pups subjected to perinatal hypothyroidism. The HDAC inhibitor could restore TH-regulated genes such as *BDNF*, *MBP*, and *PCP2* in the cerebellum and also reverse neurocognitive deficits in these rats [171]. In another study a case of pseudohypoparathyroidism in a baby girl was found to be associated with an altered methylation pattern of the *GNAS* gene, which encodes for an essential signal transduction molecule in various metabolic pathways [172]. These studies strongly suggest that there is an imbalance in the epigenetic signature, exhibiting hypothyroidism-induced abnormalities in the brain structure and functions.

## 6. Infection

The perinatal maternal environment plays a key role in the normal development and functioning of the brain. Maternal infections caused by viruses, bacteria, or even parasitic protozoans have been associated with neurodevelopmental disorders and cognitive impairments. It is possible that maternal infection alters the epigenetic mechanisms that regulate developmental control genes and signaling pathways vital for normal fetal development, leading to neurological outcomes in offspring. Recently, abnormal inflammatory cytokine secretion due to maternal infection (such as hematogenous infection with genital mycoplasma) has been found to alter the balance between pro-inflammatory and anti-inflammatory genes, leading to abnormal brain development and autism [173,174,175,176]. Maternal inflammation has further been found to alter serotonin synthesis from tryptophan within the placenta, leading to altered serotonergic axonal growth and aberrant brain development as well as autism [177,178]. Recent studies further suggest that virus interaction with Toll-like receptors (TLRs) inhibits neuronal stem cell proliferation in the neocortex, leading to behavioral dysfunction [179].

The fetal immune system is incompetent and more susceptible to infection via vertical transmission. Maternal chikungunya virus (CHIKV) infection is related to neonatal mortality and various complications including neurocognitive impairments and meningoencephalitis [180,181,182]. More recently, maternal zika virus (ZIKV) infection has been associated with microcephaly and several other neurodevelopmental disorders such as lissencephaly, hydrocephaly, ventriculomegaly, cerebellar hypoplasia, brain calcifications, and posterior fossa destruction as well as opthalamologic alterations, arthrogryposis, and spontaneous abortions. These neurodevelopmental disorders cause aberrant and delayed development, psychomotor retardation, intellectual disability, and even mortality in infants [183,184,185,186]. Human immunodeficiency virus (HIV) infection in newborns, which is acquired during pregnancy, is linked with serious neurological problems. Although preventive measures are taken to prevent HIV infection in the fetus, perinatal exposure to HIV causes altered white matter microstructural integrity in uninfected neonates [187,188,189,190]. In addition, maternal hepatitis B and hepatitis C infection are associated with neurological outcomes [191]. Coxsackie virus infection in*-*utero is linked with neurodevelopmental abnormalities in the newborn [192]. There is also a strong association observed between maternal sexually transmitted diseases and schizophrenia in newborns [193]. 

Epigenetic modifications, such as DNA methylation, histone modifications, and small non-coding RNAs, of key developmental genes play a critical role in future disease onset and progression. DNA hypomethylation at promoter regions of MeCP2, which is involved in the downstream expression of long interspersed element 1 (*LINE1*) in the dopaminergic striatum and hypothalamus, is linked to schizophrenia in the fetus [194]. Maternal immune activation due to infection epigenetically impairs gamma-aminobutyric acid (GABA) synthesis via increased 5-methyl cytosine (5-mC) and 5-hydroxymethyl cytosine (5-hmc) modifications at *GAD1* and increased 5-mC levels at *GAD2* promoter regions in*-*utero*,* leading to altered synaptic responses in early life. This hypermethylation at GAD1 and GAD2 promoter regions is associated with impaired cognitive and social responses [195]. Babies with autism-associated copy number variation (CNVs) are more likely to develop autism if they are exposed to maternal infection in*-*utero [156]. Furthermore, the maternal auto-antibodies profile, which is epigenetically altered by microbial infection, can be adaptive, which may form the basis for autism development in early life [196]. Recent studies show that long non-coding RNAs (lncRNAs) are differentially expressed and regulate various infection and inflammatory pathways, leading to spontaneous abortions [197,198].

In summary, in*-*utero exposure to maternal infection leads to stable epigenetic changes in the fetal epigenome, causing alterations in the expression levels of genes involved in neurological diseases. Understanding the underlying epigenetic and molecular mechanism of neurological diseases altered by maternal infection in the fetus would help in overcoming some major challenges in clinical care as there is a lag time between maternal infection, neurological pathogenesis of fetal infection, and the clinical detection of vertical transmission [199,200].

## 7. Genetic and Epigenetic Regulation of Neurodevelopmental Disorders 

There are a number of genetic disorders that have been found to be epigenetically linked. The transgenerational inheritance of epigenetic traits, also termed epimutation, has been reported to play critical roles in the pathology of certain genetic disorders such as Prader-Willi and Angelman syndromes [201]. Several studies reported that Angelman, Rett, and Fragile-X syndromes, which affect the neurodevelopment, are epigenetically associated with the genotype–phenotype interactions [202,203,204]. The accelerated aging in Down’s syndrome children is also linked to their “epigenetic clock”, with an increasing load of DNA methylation [205]. Angelman syndrome (AS) is a neurogenetic disorder caused by a deletion or mutation in chromosome15 (15q11–q13) encoding for the maternally inherited E3 ubiquitin ligase *UBE3A* gene, which is essential for synapse formation and remodeling. Approximately 6% of total AS cases are caused by an imprinting defect [206], an epigenetic phenomenon regulated by differentially methylated regions in several imprinting genes. Further investigation of the epigenetic regulation of the process of genetic imprinting will help in the treatment of AS. There is a possibility of overcoming the loss of UBE3A protein by epigenetically activating the silenced parental allelic form by means of epidrugs in AS patients.

Rett syndrome, which is a genetic disorder characterized by a mutation in chromosome X (Xq28) encoding for the MeCP2 protein, is a neurodevelopmental disorder that leads to severe behavioral and physiological symptoms. MeCP2 is a protein containing a methyl CpG binding domain (MBD), which is associated with transcriptional regulation by DNA methylation. Missense mutations in the MBD of MeCP2 gene are associated with Rett syndrome [207]. A recent case study proposed that an inversion in the X chromosome may cause anomalies in genomic interactions, which alter the epigenetic mechanisms and lead to MeCP2-associated Rett syndrome [203]. Loss of MeCP2 in a mouse model has been shown to be associated with aberrant long non-coding RNAs (lncRNAs) and miRNAs expression, contributing to Rett syndrome [208]. Recently, the successful derivation of iPSC lines from Rett patients opens up the avenue to elucidate novel therapeutics to reverse the epigenetic insults linked to the MeCP2 mutation [209].

Fragile-X syndrome (FXS) is another inherited disorder where fragile X mental retardation 1 (*FMR1*) gene silencing leads to mental retardation and autism in males. *FMR1* regulates several neurotransmitters important for synaptic functions [210]. The epigenetic silencing of the X-linked *FMR1* gene has been shown to result in FXS pathophysiology. An increased level of DNA methylation (5mC) and hydroxylmethylation (5hmC) at the transcriptional start site of *FMR1* in FXS patients highlights the strong association of epigenetic regulation in *FMR1* gene silencing [204]. Hence epigenetic therapeutic strategies using DNA demethylating agents at the transcriptional site to reactivate *FMR1* offer promising avenues to treat this currently non-treatable genetic disorder [211]. 

## 8. Conclusions

**Figure 5:**
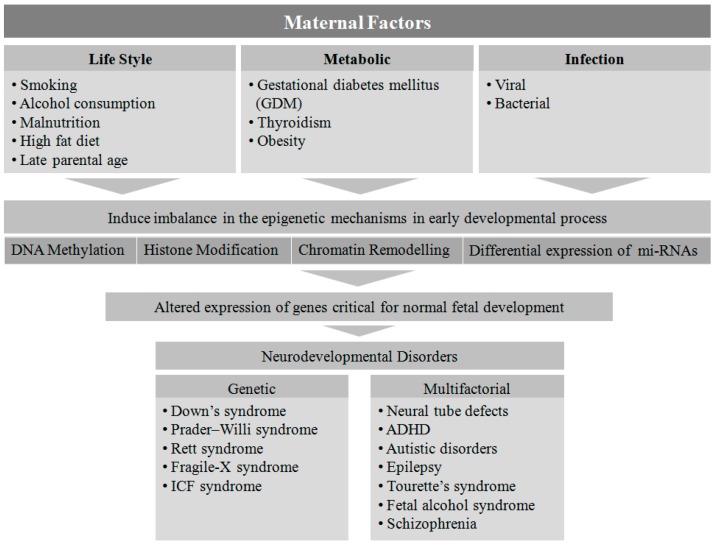
Maternal factor-induced epigenetic etiology for neurodevelopmental disorders. Several lifestyle-related metabolic factors and infection at gestation play a critical role in the epigenetic modification and in turn the altered expression of many genes associated with abnormal fetal development. This may lead to a series of neurodevelopmental disorders in the offspring.

From the above discussion it is clear that maternal lifestyle factors such as smoking, drinking, diet, gestational age, metabolic disorders, and infection during pregnancy play a significant role in the development of NDs in offspring (Figure 5). The epigenetic link to the etiology of NDs is well demonstrated through numerous pre-clinical and clinical cohort studies. There is a great deal of evidence showing a widespread modulation in the DNA methylation patterns along with histone modification and miRNA mediated gene regulation towards the pathophysiology of NDs. Because of the plasticity of epigenetic regulations, which can be reversed by early interventions, the role of epigenetic regulators in the treatment strategy of NDs is a promising platform to investigate further. Although the research on epidrug discovery is still in its infancy, it is rapidly growing as an epicenter for the management of these developmental disease conditions, which are otherwise irreversible by current therapeutic modalities [212]. This will open up new vistas of opportunity for developing new diagnostic tools based on the unique epigenetic signatures in these disorders [213].

There are several histone-modifying enzyme inhibitors currently under investigation to develop into feasible therapeutic targets [12,214]. HDAC inhibitors such as valproic acid have already been found to be effective in the management of several psychiatric disorders including epilepsy and ASD [185]. Valproic acid is an FDA-approved drug used globally for the management of mood disorders and epileptic seizures [215]. A few other HDAC inhibitors have also reached the level of clinical trials to test their safety and efficacy in various neuropsychological disorders (ClinicalTrials.gov Identifier: NCT02654405; NCT02094651) [216,217]. The DNA methylation machinery is another intrinsic factor contributing to the pathology of NDs. Deficiency in several dietary micronutrients such as folate and choline during pregnancy has shown a direct correlation with the development of congenital abnormalities by altered DNA methylation through the FOCM pathway [17]. Furthermore, it is reported in a large prospective mother-children cohort that the maternal diet during pregnancy has a predictive role in the subsequent neuropsychological health of the offspring [218]. Hence, early intervention by supplementing the maternal diet with DNA methylating agents at gestation would be helpful in maintaining the healthy development of the fetus [219].

In the last two decades, the scientific community has collated critical information on the epigenetic factors associated with NDs, including the pattern of DNA methylation, histone modification, and miRNA-based regulation of gene expression. However, the mechanism by which epigenetic factors influence the expression pattern of genes and subsequent fetal phenotypes is extremely complex. Further comprehensive longitudinal studies need to be undertaken to unravel the pathways linking maternal lifestyle and the fetal developmental cascade involving epigenetic and molecular changes. A better understanding of this nascent field will not only take us a step forward towards the realization of epigenetic medicine for the management of neuropsychiatric disorders, but also mitigate their toll on society.
